# Optimization of Microwave-Assisted Extraction of Antioxidants from Bamboo Shoots of *Phyllostachys pubescens*

**DOI:** 10.3390/molecules25010215

**Published:** 2020-01-05

**Authors:** Gualtiero Milani, Francesca Curci, Maria Maddalena Cavalluzzi, Pasquale Crupi, Isabella Pisano, Giovanni Lentini, Maria Lisa Clodoveo, Carlo Franchini, Filomena Corbo

**Affiliations:** 1 Department of Pharmacy-Pharmaceutical Sciences, University Aldo Moro-Bari, Via Orabona, 4, 70126 Bari, Italy; gualtiero.milani@uniba.it (G.M.); francesca.curci@uniba.it (F.C.); giovanni.lentini@uniba.it (G.L.); carlo.franchini@uniba.it (C.F.); filomena.corbo@uniba.it (F.C.); 2 CREA-VE, Council for Agricultural Research and Economics—Research Centre for Viticulture and Enology, Via Casamassima, 148, 70010 Turi, Italy; pasquale.crupi@crea.gov.it; 3 Department of Bioscience, Biotechnology and Biopharmaceutics, University of Bari, Via Orabona, 4, 70125 Bari, Italy; isabella.pisano@uniba.it; 4 Interdisciplinary Department of Medicine, University of Bari, Piazza Giulio Cesare, 11, 70124 Bari, Italy; marialisa.clodoveo@uniba.it

**Keywords:** bamboo, microwave-assisted extraction, Folin-Ciocalteu, antioxidant activity, design of experiments

## Abstract

Bamboo is a well-known medicinal plant in Southeast Asia that recently has attracted attention for its high polyphenol content and its medical and nutraceutical applications. In this work, polyphenols have been recovered for the first time by microwave-assisted extraction (MAE) from an unusual Italian cultivar of *Phyllostachys pubescens* bamboo shoots. The effects of three independent variables, such as extraction time, temperature, and solid/liquid ratio, on polyphenol recovery yield were investigated and successfully optimized through the response surface methodology. We demonstrated that MAE is an excellent polyphenols extraction technique from bamboo shoots because the total phenolic content obtained under microwave irradiation optimal conditions (4 min at 105 °C with 6.25 mg/mL ratio) was about eight-fold higher than that obtained with the conventional extraction method. Furthermore, higher total flavonoid content was also obtained under MAE. Consistent with these results, MAE enhanced the extract antioxidant properties with significant improved DPPH, ABTS, and FRAP scavenging ability. Therefore, this innovative extraction process enhances the recovery of biologically active compounds from *Phyllostachys pubescens* bamboo shoots with a dramatic reduction of time and energy consumption, which paves the way for its industrial application in functional food production.

## 1. Introduction

Bamboo is a perennial, woody grass, evergreen plant, and one of the oldest plants on Earth. Although more than 1250 species belonging to 75 genera are distributed worldwide, it grows predominantly in Asia [1,2]. Bamboo is popularly known for its industrial uses and has long been used in China and Southeast Asia both as a food and in traditional medicine [3]. In fact, several pharmacological properties have been reported for bamboo such as anticancer, antioxidant, antibacterial, antidiabetic, antiulcer, anti-inflammatory, and antihypertensive activities [2,4]. Angiotensin-converting enzyme inhibition activity has also been recently proposed [5]. Although most research studies have investigated the functional activities of bamboo leaves [6,7,8,9,10,11,12,13,14,15,16], only a few studies have been reported on the functional properties of bamboo shoots, which rank among the five most popular healthcare foods in the world. They are not only delicious but also rich in some nutrient components, such as vitamins, proteins, carbohydrates, and minerals. However, on the other hand, they have low fat content [1,3,17,18].

In particular, they show high content of secondary metabolites—such as phenolic compounds, flavones, and glycosides—which play a pivotal role in providing protection against many chronic and degenerative diseases [19,20]. Among them, phenolic compounds, which are commonly referred to as polyphenols, have been reported to be responsible for the antioxidant and anti-inflammatory activities of bamboo shoot extracts [5,21,22,23]. In fact, it is well known that polyphenols are involved in the redox balance [19] as in the regulation of immune responses [24] of the cells. These features make polyphenols a potentially interesting material for the development of functional foods [25]. Recently, Park et al., reported on the phenolic compound composition and antioxidant activity of different extracts of *Phyllostachis pubescens* shoots [5]. It is the major species of bamboo in Japan, China, and Korea, belonging to the family *Poaceae* (or *Gramineae*) and subfamily *Bambusoideae* [26]. Since conventional extraction methods, such as that used by Park et al., require long extraction times [27], we decided to optimize the extraction procedure applying a novel extraction technology. Among the innovative technologies, microwave-assisted extraction (MAE) has gained more attention in the past decade due to its advantages such as shortened extraction time, reduced solvent consumption, higher extraction rate, and lower energy consumption [28,29]. Moreover, the high reproducibility as well as the controlled temperature and pressure are considered strengths of MAE, which has been extensively used to secondary metabolite extraction from different plant matrices [30,31,32]. Thus, we chose this approach not only for the many advantages it offers but also because no one had ever been investigated before the effects of microwaves on the polyphenol extraction from bamboo shoots of *Phyllostachys pubescens*. In particular, an unusual Italian cultivar of *Phyllostachys pubescens* has been used for the first time with the aim of evaluating the possibility of extraordinarily promoting the cultivation of this plant in the Italian country. Our goal was to demonstrate that microwave irradiation was able to enhance the polyphenol content of bamboo shoot extracts, which perform better with respect to conventional solid-liquid extraction. It is well known that microwave irradiation aids plant matrix disruption and, together with a high temperature extraction that reduces the solvent viscosity, allows for greater recovery of polyphenols and flavonoids. On the other hand, shorter extraction time, in turn, reduces the risk of decomposition and oxidation of phytochemicals, which often ensures high TPC values [33].

Since many parameters could affect the MAE process, it would be advisable to use a statistical technique for quickly optimizing the extraction procedure. Therefore, we used the response surface methodology (RSM) to analyze the influence of many parameters simultaneously as well as their interactions on the response so that the optimal experimental conditions can be readily and confidently established. Extraction time, temperature, and the solid/liquid ratio (mg/mL) have been chosen as variables to be optimized so as to obtain the highest phenolic recovery. Furthermore, the flavonoid content and antioxidant activity of the extracts obtained from both conventional and optimized microwave-assisted procedures were also evaluated.

## 2. Results

### 2.1. Conventional Extraction Procedure

At first, we performed the bamboo shoot conventional extraction, according to the procedure recently proposed by Park et al. [5], which maintains the same relationship between the bamboo shoots and solvent (50 mg/7.5 mL). In these conditions, we obtained 11.2 ± 0.1 mg GAE/g of bamboo shoots as a TPC value. Having planned to improve the polyphenol extraction with the aid of microwave irradiation, it notoriously allows reducing the solvent consumption. We decided to use 2 mL as the starting volume in the next MAE (see Supplementary Material). However, it was necessary to first carry out the conventional extraction in the same volume (50 mg/2 mL) to, hereafter, compare conventional results and MAE results. Having observed a low reduction in the TPC (6.6 ± 0.1 mg GAE/g of bamboo shoots), and hoping to significantly increase it with the aid of RSM, these conditions represented our starting point for the next MAE.

### 2.2. Optimization of the Phenolic Extraction Yield under MAE by RSM

Among the most widely used solvents for extracting phenolic substances, we first chose methanol according to the procedure proposed by Park et al. [5]. Our goal was to enhance their conventional phenolic extraction yield by taking advantage of the use of microwave irradiation, without further modifying their experimental conditions.

The MAE processing conditions selected as independent variables of the experimental design aimed at increasing the extraction of polyphenols from bamboo shoots (*Phyllostachys pubescens*) were extraction time (5–10 min), temperature (58–68 °C), and solid/liquid ratio (12.5–25 mg/mL). This preliminary study showed that higher temperature and both lower concentration and extraction time were necessary to optimize our extraction process (see Supplementary Material). Therefore, RSM was applied for modelling and predicting total phenolic content (TPC) yield and, according to the CCF (central composite face-centered) design, 17 runs were performed for the optimization of the three chosen parameters. The design matrix and the corresponding response values (TPC) were listed in Table 1.

The quadratic equation derived for TPC is given in Equation (1).

Y = 0.781163 + 0.264907X_1_ − 0.0794X_2_ + 0.0244X_3_ + 0.424X_1_^2^ − 0.033535X_2_^2^ − 0.034535X_3_^2^ + 0.010125X_1_X_2_ + 0.038625X_1_X_3_ + 0.030375X_2_X_3_ (1)

where X_1_ is the extraction temperature, X_2_ is the solid/liquid ratio, and X_3_ is the extraction time.

It is evident that the response value was mostly affected by the extraction temperature (X_1_) as shown by the linear and quadratic terms of the equation. To a lesser extent, the liquid/solid ratio (X_2_) also affected the response value. The extraction time (X_3_) is almost irrelevant. The results suggest that the change of extraction temperature had highly significant effects on the yield of polyphenols (*p* < 0.0001) from the bamboo shoots of *Phyllostachys pubescens* under microwave irradiation.

By the analysis of variance (ANOVA) of a simpler second-order polynomial regression model obtained by omitting insignificant terms, the value of the determination coefficient (R^2^) and adjusted determination coefficient (R^2^_Adj_) were 0.943 and 0.929, respectively, which suggests good agreement between the observed and predicted values. Furthermore, the high value of cross-validated determination coefficients (Q^2^ = 0.883) indicated a good predictive power of the model. On the other hand, the regression model was statistically relevant, with a significance level lower than 0.0001 (*p* < 0.0001), and had no significant lack of fit (*p* = 0.112). Taken together, these results confirm that a well-fitting model has been obtained.

**Figure 1:**
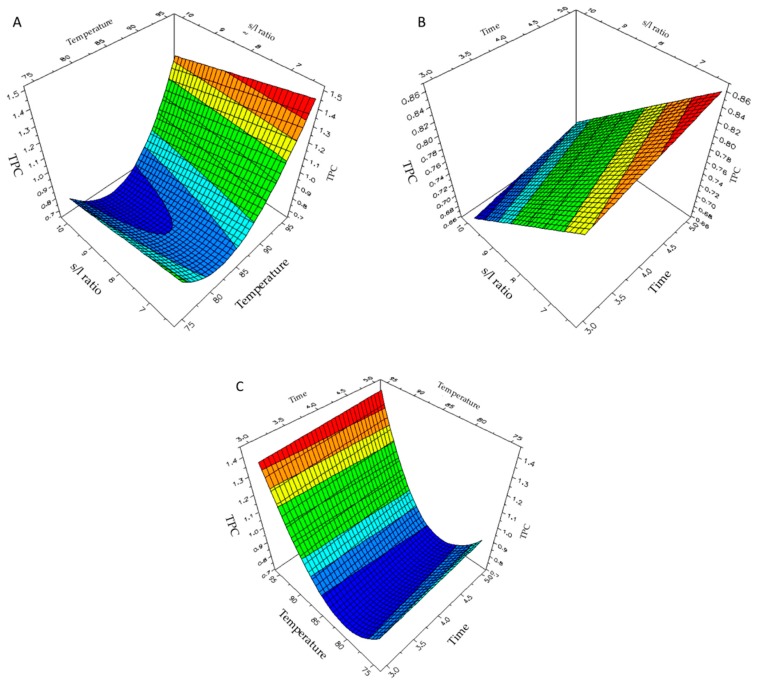
Response surface plots of the effect of (**A**) solid/liquid ratio and extraction temperature, (**B**) solid/liquid ratio and extraction time, (**C**) extraction temperature and time on polyphenol yield (TPC) obtained through MAE.

Three-dimensional response surface plots, generated by the model, and reported in Figure 1, indicated the effect of each variable on the polyphenol yield (TPC). For the sake of clarity, two variables were represented within the surface plot experimental plane while the other was kept constant at its center level.

An increase in the extraction temperature (see Figure 1A,C) and a concomitant reduction of sample concentration (see Figure 1A) enhanced the extraction yield. As expected from the equation coefficients, extraction time did not strongly affect the response variable and it was almost irrelevant, in the selected range of 3–5 min, compared to the extraction temperature (see Figure 1C).

The found temperature influence is in agreement with the well-known improvement of extraction yield in MAE attributed to microwave heating that occurs by both dipolar polarization and ionic conduction, which are typical of polar and polarizable substances. In fact, as a result of dipole rotation of molecules, hydrogen bonds are broken and the solvent diffusion into the matrix is considerably improved, with the extraction yield being enhanced [34]. The water present or the solvent penetrated into plant cells absorb microwave energy, which causes an internal superheating. This can disrupt membrane cells, which facilitates leaching out of the active constituents into the solvent and improving the polyphenol recovery [35]. This phenomenon is greater when the extraction is carried out with solvents with higher heating efficiency under the microwave (higher tan δ value), as for MeOH [36]. On the other hand, prolonged extraction time at high temperatures could cause the degradation or oxidation of thermolabile phytochemicals [37], which can be avoided through the shorter extraction time guaranteed by MAE. According to this knowledge, when RSM was applied to bamboo shoots, MAE indicated a particularly short extraction time of just 4 min, which supports the validity of our model. Based on the results presented in Table 1, we can state that higher temperature applied for only a few minutes exerted a significantly positive influence on the amounts of TPC extracted using MAE, even though more in-depth studies are needed to ascertain that there has been no degradation of the biologically-active compounds.

Based on the RSM obtained results, the values predicted by the software as the optimal conditions under microwave irradiation were: 6.25 mg/mL under microwave irradiation for 4 min at 105 °C and the predicted TPC was 2.9 ± 0.3 mg gallic acid equivalent (GAE). Under these conditions, the experimental value was 2.74 ± 0.08 mg of GAE (corresponding to 54.8 ± 1.6 mg GAE/g dry weight, entry 3-Table 2), which is much higher than that obtained for both the conventional extracts, regardless of the solvent volume used [7.5 mL (entry 1-Table 2) or 2 mL (entry 2-Table 2) for 50 mg of bamboo shoots]. Hence, we can state that the MAE under optimal conditions allowed us to optimize the extraction process proposed by Park et al. [5].

On the other hand, our results pave the way for industrial application of bamboo shoots (*Phyllostachys pubescens*) MAE since our extracts, with high antioxidant content and obtained in a few minutes (thus, reducing energy consumption), could serve as valuable sources of natural antioxidants in the nutraceutical and cosmetic industries. This will be achievable when extractions will be performed with a green solvent (extractions with water or EtOH are under investigation).

### 2.3. Total Flavonoid Content

The total flavonoid content in the bamboo shoot extracts obtained with both extraction methods were also determined and the values obtained are 0.81 ± 0.01 mg QE/g dry weight and 1.3 ± 0.1 mg QE/g dry weight in conventional and microwave-assisted extracts, respectively (Table 2). Therefore, microwave irradiation undoubtedly also improves the number of flavonoids extracted from bamboo shoots, which is about twice that obtained by conventional extraction.

### 2.4. Antioxidant Activity of the Extracts Obtained from Bamboo Shoots either by Conventional Extraction or at Optimal MAE Conditions

Several methods can be employed to evaluate the in vitro antioxidant activity of plant extracts and we chose DPPH, ABTS, and FRAP assays, which are among the most widely used. The results for the extracts obtained with both reflux extraction and MAE at optimal conditions are reported in Table 2.

The obtained results demonstrated that the MAE at the optimal conditions (105 °C, 4 min, and 6.25 mg bamboo shoots/mL methanol) showed higher efficiency of antioxidant extraction compared to the conventional extraction method (room temperature, 24 h, 25 mg bamboo shoots/mL methanol). In fact, the antioxidant activity observed with the DPPH assay was more than twice higher in the microwave extracts and in both ABTS and FRAP assays of the mg Trolox equivalent (TE)/g dry weight was even 8–20-fold higher in the extracts obtained through the MAE process with respect to the conventional method. These results underline the usefulness of microwave irradiation as an innovative extraction procedure applied to bamboo shoots of *Phyllostachys pubescens* since it guarantees a higher recovery of an antioxidant compound in extremely reduced times. In fact, the microwave-assisted extraction, which gave the results reported in Table 2, has been performed in only 4 min with a significant reduction in energy consumption and a considerable gain in time.

### 2.5. HPLC Analyses

In order to corroborate our previous observation about the antioxidant capacity, HPLC-DAD analyses of the extracts of bamboo shoots, obtained under optimal conditions, were performed. As depicted in Figure S2, the chromatographic profiles seem to confirm the found TPC and TFC values. The MW extract (A) was richer of polyphenolic compounds, especially those absorbing at 320 nm (Figure S2b), than a conventional one (B). Polyphenols revealed at 320 nm generally belongs to the family of hydroxycinnamic acids (i.e., caffeic acid, chlorogenic acid, and *p*-coumaric acid) or flavones (i.e., apigenin and luteolin glycosides), already identified in bamboo extracts [38,39], that are characterized by antioxidant properties [40]. Therefore, the consistent difference in their content between the two extracts (A vs. B) could be related to the higher antioxidant capacity of the MW extract. Anyway, further analyses involving mass spectrometry acquisitions will need to correctly identify and quantify these compounds.

## 3. Materials and Methods

### 3.1. Plant Material

Fresh bamboo shoots (500 g) of *P. pubescens* were harvested on April 2016 from Ravenna cultivar (Emilia Romagna, Italy) and provided by Consorzio Bamboo Italia. They were collected and kept at −20 °C until needed. Leaves were removed and bamboo shoots were sliced and freeze-dried (Christ Alpha 1-4 LSC). Dried bamboo shoots were then finely grinded with mortar and pestle and sifted through mesh strainers (200–500 μm, Cecchinato).

### 3.2. Chemicals and Reagents

MeOH used for extraction, Folin-Ciocalteu reagent, gallic acid, acetic acid, sodium carbonate, quercetin, aluminum chloride (AlCl_3_), sodium-potassium tartrate, 2,2-diphenyl-1-picrylhydrazyl (DPPH), 2,2′-azobis(2-amidinopropane) dihydrochloride (AAPH), 2,4,6-tris(2-pyridyl)-*s*-triazine 98%, and 6-hydroxy-2,5,7,8-tetramethyl chromane 2-carboxylic acid (trolox) were purchased from Sigma-Aldrich (Milan, Italy). 2,2′-Azinobis(3-ethylbenzothiazoline-6-sulphonic acid)diammonium salt (ABTS), iron chloride hexahydrate, and sodium acetate trihydrate 99% were purchased from Alfa Aesar (Karlsruhe, Germany). Formic acid, acetonitrile, and water HPLC grade used for HPLC analyses were purchased from Sigma Aldrich (Milano, Italy).

### 3.3. Conventional Extraction

A suspension of 50 mg of bamboo shoot powder in the appropriate volume of MeOH (7.5 or 2 mL, entries 1, 2 of Table 2) was stirred at room temperature for 24 h. The solution was then filtered through Whatman (No. 1) filter paper and stored at −20 °C until needed for analysis.

### 3.4. Microwave-Assisted Extraction (MAE)

A closed-system MAE was carried out at constant temperature, with continuous stirring, in a CEM Discover Bench Mate microwave reactor equipped with Synergy software. The temperature was measured and controlled by a built-in infrared detector. Briefly, 50 mg of bamboo shoot powder in the appropriate volume of solvent, according to the solid/liquid ratio (mg/mL) value reported in Table 1, were irradiated with microwaves (35 W) at different temperatures for different times (see Table 1). The solution was then filtered through Whatman (No. 1) filter paper and stored at −20 °C until needed for analysis.

### 3.5. Polyphenol Assays

#### 3.5.1. Determination of the Total Phenolic Content (TPC)

The total amount of polyphenols in the prepared extracts was determined according to the method described by Blainski [41], with some modification. The Folin-Ciocalteu reagent was used and a standard calibration curve (R^2^ = 0.9987) was prepared using different concentrations of gallic acid in methanol (0.025–0.200 mg/mL). Furthermore, 50 µL aliquot of each sample, 50 µL of Folin-Ciocalteu reagent, 50 µL of MeOH, and 250 µL of water were mixed. In addition, 200 µL of sodium carbonate (20%) and 400 µL of water were then added and the solution was incubated at 30 °C for 90 min after which its absorbance was measured against a reagent blank at 700 nm (Perkin Elmer, Lambda Bio 20, Boston, MA, USA). The assay was performed in triplicate and the final results were expressed as milligrams of a gallic acid equivalent (GAE) per g of bamboo shoots (mg GAE/g of bamboo shoots).

#### 3.5.2. Determination of the Total Flavonoids

The total amount of flavonoids was determined according to the aluminum chloride method described by Khatiwora et al. [42]. A calibration curve (R^2^ = 0.9983) was prepared using different concentrations of quercetin in methanol (2.5–18 μg/mL). Afterward, 16 µL of AlCl_3_ (10%) and 16 µL of Na^+^/K^+^ tartrate (10%) were added to 500 µL aliquot of each sample. The solution was then brought to 1 mL with water and incubated for 30 min. The absorbance was measured at 415 nm (Perkin Elmer, Lambda Bio 20) against a reagent blank. The assay was performed in triplicate and the results were expressed as mg of quercetin equivalent (QE) per g of bamboo shoots (mg QE/g of bamboo).

### 3.6. Antioxidant Activity

#### 3.6.1. DPPH Assay

Total free radical scavenging capacity of the extracts using the stable DPPH radical was determined according to the previously reported procedure [43,44], with some modifications. A calibration curve (R^2^ = 0.9934) was prepared using different concentrations of gallic acid in methanol (0–4 μg/mL). In addition, 650 μL of each sample were diluted with 350 μL of a methanolic solution of a DPPH radical to obtain a final concentration of 100 μM. The solution was then vigorously mixed and incubated at 30 °C for 30 min in the dark. The reduction of DPPH concentration was recorded by a decrease in absorbance at 517 nm using a UV-vis spectrophotometer (Perkin Elmer, Lambda Bio 20) against a reagent blank and the assay was performed in triplicate. The results were expressed as SC_50_ values (μg/mL necessary for 50% reduction of the DPPH radical), which were calculated with the calibration curve obtained with different concentrations of a sample extract.

#### 3.6.2. ABTS Assay

The antioxidant activity of the extracts using ABTS radical cation decolorization assay was determined, according to the procedure previously reported by Arnao et al. [45], with some modifications. The stock solutions included 7.4 mM ABTS^•+^ solution and 2.6 mM potassium persulfate solution. The working solution was then prepared by mixing the two stock solutions in equal quantities and allows them to react for 12 h at room temperature in the dark. The solution was then diluted by mixing 1 mL ABTS^•+^ solution with 30 mL methanol to obtain an absorbance of 1.17 ± 0.02 units at 734 nm using the spectrophotometer. Fresh ABTS^•+^ solution was prepared for each assay. The standard curve (R^2^ = 0.9981) was linear in the range of 0 to 150 µg/mL Trolox. The extracts (150 mL) were allowed to react with 2850 mL of the ABTS^•+^ solution for 10 min in a dark condition. Then the absorbance was taken at 734 nm using the spectrophotometer. Results are expressed in mg Trolox equivalents (TE) per g of bamboo shoots (mg TE/g bamboo shoots) and were calculated using the following formula.

$$ABTS\;radical\;scavenging\;activity=\frac{Absorabnce_{blank}-Absorbance_{sample}}{Absorbance_{blank}}\times 100$$

Additional dilution was needed if the ABTS value measured was over the linear range of the standard curve.

#### 3.6.3. FRAP Assay

The antioxidant capacity of the extracts was measured spectrophotometrically through the FRAP assay following the procedure of Benzie and Strain [46], with some modifications. The stock solution was prepared with 300 mM acetate buffer (3.1 g CH_3_CO_2_Na·3H_2_O and 16 mL CH_3_COOH), 10 mM TPTZ (2,4,6-tripyridyl-*s*-triazine) solution in 40 mM HCl, and 20 mM FeCl_3_·6H_2_O solution. The fresh working solution was prepared by mixing 25 mL of acetate buffer, 2.5 mL of TPTZ solution, and 2.5 mL of FeCl_3_·6H_2_O solution, and then warmed at 37 °C before using. The extracts (150 μL) were allowed to react with 2850 μL of the FRAP solution for 30 min in the dark condition. Readings of the colored product [ferrous tripyridyltriazine complex] were then measured at 593 nm. The standard curve (R^2^ = 0.9962) was linear in the range 0–200 µg/mL Trolox. Results are expressed in mg TE/g dry weight. Additional dilution was needed if the FRAP value measured was over the linear range of the standard curve.

### 3.7. Experimental Design for Microwave Extraction Optimization (RSM)

The extraction parameters were optimized using response surface methodology. A central composite face-centered design (CCF) was employed to evaluate the effects of three independent parameters [extraction temperature (X_1_), solid/liquid ratio (X_2_), and extraction time (X_3_)] on the response variable (TPC), as shown in Table 1. The values of the three independent variables reported in Table 1 were based on the results of preliminary experiments (data not shown). Seventeen experiments were conducted, with three replications at the center values to evaluate the pure error sum of squares. Experimental data were fitted with the quadratic model shown below (Equation (2)).

*Y* = *b*_0_ + ∑*b_i_X_i_* + ∑*b_ii_X_i_*^2^ + ∑b_*ij*_*X_i_X_j_* (2)

where *Y* is the dependent parameter (TPC), *X_i_* and *X_j_* are the independent variables (*X*_1_–*X*_3_), b_0_ is the intercept, and *b_i_*, *b_ii_*, and *b_ij_* are the linear, quadratic, and interaction coefficients, respectively. The model prediction capability is commonly explained by the coefficient of determination (R^2^). The analysis of variances (ANOVA) was also used to evaluate the quality of the fitted model. The test of statistical difference was based on the total error criterion with a confidence level of 95%. Analysis of the experimental design data and calculation of predicted responses were carried out using the commercial Modde 6.0 software (Umetrics, Malmo, Sweden).

### 3.8. HPLC-DAD Analyses

HPLC-DAD analysis was performed by an HPLC 1100 (Agilent Technologies, Palo Alto, CA, USA) equipped with a degasser, quaternary pump solvent delivery, thermo-stated column compartment, and a diode array detector. Chromatographic separation was performed by using Zorbax SB C18 3.5 µm (150 × 4.6 mm i.d., Agilent Technologies) with a pre-column Gemini C18 5 µm (4 × 2 mm i.d., Phenomenex) as reversed stationary phase, and a binary solvent system (solvent A: 1% formic acid in water, solvent B: acetonitrile) as a mobile phase. The following gradient elution program was used: 0–1.5 min, 2% B, 1.5–3 min, 2–9% B, 3–6 min, 9–14% B, 6–7 min, 14–19% B, 7–8 min held at 19% B, 8–15 min, 19–27% B, 15–25 min, 27–37% B, 25–30 min, 37–90% B, 30–32 min, held at 90% B, 32–33 min, 90–2% B, held for 5 min at 2% B to re-equilibrate the column. Flow rate, column temperature, and injection volume were set up at 1.0 mL min^−1^, 40 °C, and 3 µL. Diode array detection was between 250 and 600 nm, and absorbances were recorded at 280 and 320 nm.

## 4. Conclusions

In this study, response surface methodology has been applied to optimize the TPC in bamboo shoot (*P. pubescens*) extracts obtained by MAE. Influencing parameters on the extraction yield of polyphenols were studied and the optimal found MAE conditions predicted by the quadratic model generated by RSM design were solid/liquid ratio of 6.25:1 (mg/mL), microwave extraction temperature of 105 °C, and microwave extraction time of 4 min that gave 54.8 ± 1.6 mg GAE/g dry weight as TPC content. This result demonstrates the usefulness of microwave irradiation for the polyphenol extraction from bamboo shoots since the TPC value obtained with microwave irradiation was about eight-fold higher than that obtained with the conventional extraction. Although a conventional extraction has not been carried out at the same temperature, which would have been necessary to compare the effects of conductive and dielectric heating, it can be stated that the use of microwaves guaranteed at least a drastic reduction in extraction time (to only 4 min). Thus, at the laboratory scale, MAE performs as a powerful method for increasing extraction efficiency of antioxidant compounds from bamboo shoots of *P. pubescens* reducing time and energy consumption. On the other hand, the scale-up of our method is necessary to ensure the economical industry production of the highest quality extracts from bamboo shoots, which faces the ever-increasing need for natural anti-oxidant agents.

## Figures and Tables

**Table 1:** Optimization of microwave-assisted extraction of bamboo shoots using response surface methodology: central composite face-centered (CCF) design matrix and observed responses.

Experiment No.	Extraction Temperature (°C)	Solid/Liquid Ratio (mg/mL)	Extraction Time (min)	TPC ^a^
1	75	6.25	3	0.946
2	95	6.25	3	1.423
3	75	10	3	0.796
4	95	10	3	1.239
5	75	6.25	5	0.910
6	95	6.25	5	1.467
7	75	10	5	0.807
8	95	10	5	1.479
9	75	8.12	4	0.970
10	95	8.12	4	1.009
11	85	6.25	4	0.947
12	85	10	4	0.578
13	85	8.12	3	0.769
14	85	8.12	5	0.754
15	85	8.12	4	0.733
16	85	8.12	4	0.761
17	85	8.12	4	0.790

^a^ expressed as mg GAE in the total extraction volume.

**Table 2:** Total phenolic, flavonoid contents, and antioxidant activity of bamboo shoot extracts obtained under two different extraction methods.

Entry	Extraction Method	Time	TPC ^a^	TFC ^b^	DPPH ^c^	ABTS ^d^	FRAP ^d^
1	Conventional ^e^	24 h	11.2 ± 0.1	-	-	-	-
2	Conventional ^f^	24 h	6.6 ± 0.1	0.81 ± 0.01	23.33	9.1 ± 0.1	3.22 ± 0.01
3	MAE ^f^	4 min	54.8 ± 1.6	1.3 ± 0.1	9.20	71.5 ± 0.1	62.15 ± 0.01

^a^ Total phenolic content, expressed as mg GAE/g dry weight. Values represent means ± SD (*n* = 3). ^b^ Total flavonoid content, expressed as mg QE/g dry weight. Values represent means ± SD (*n* = 3). ^c^ SC_50_: radical scavenging activity (concentration expressed in μg/mL necessary for 50% reduction of DPPH radical). ^d^ expressed as mg TE/g dry weight. Values represent means ± SD (*n* = 3). ^e^ 50 mg/7.5 mL according to the procedure of Park et al. [5]. ^f^ 50 mg/2 mL.

## Supplementary Materials

The following are available online at https://www.mdpi.com/1420-3049/25/1/215/s1, Table S1: Screening of parameters influencing microwave-assisted extraction of bamboo shoots: design matrix and observed responses; Figure S1: Response surface plot of the effect of extraction temperature and solid/liquid ratio on polyphenol yield (TPC) obtained through MAE; Figure S2: HPLC-DAD chromatograms recorded at a) 280 nm and b) 320 nm of the bamboo shoots MW extract obtained under optimal conditions (A) and conventional extract (B), respectively.
