# Supplementary material for: Optimization of Microwave-Assisted Extraction of Antioxidants from Bamboo Shoots of Phyllostachys pubescens
Source: Molecules. 2020 Jan 5;25(1):215. doi: 10.3390/molecules25010215 (PMC6982868; doi:10.3390/molecules25010215)
Supplement: Supplementary file 1 [file molecules-25-00215-s001.pdf]

Supplementary Material

Optimization of microwave-assisted extraction of antioxidants from bamboo shoots of *Phyllostachys pubescens*

Gualtiero Milani [1], Francesca Curci [1], Maria Maddalena Cavalluzzi [1*], Pasquale Crupi [2], Isabella Pisano [3], Giovanni Lentini [1], Maria Lisa Clodoveo [4], Carlo Franchini [1], Filomena Corbo [1]

[1] Department of Pharmacy-Pharmaceutical Sciences, University Aldo Moro-Bari, Via Orabona, 4, 70126 Bari, Italy; gualtiero.milani@uniba.it (G.M.); francesca.curci@uniba.it (F.C.); mariamaddalena.cavalluzzi@uniba.it (M.M.C.); giovanni.lentini@uniba.it (G.L.); carlo.franchini@uniba.it (C.F.); filomena.corbo@uniba.it (F.C.)

[2] CREA-VE, Council for Agricultural Research and Economics – Research Centre for Viticulture and Enology, Via Casamassima, 148 – 70010 Turi (BA), Italy; pasquale.crupi@crea.gov.it (P.C.)

[3] Department of Biosciences, Biotechnologies and Biopharmaceutics, University of Bari, Via Orabona, 4, 70100 Bari, Italy; isabella.pisano@uniba.it (I.P.);

[4] Interdisciplinary Department of Medicine, University of Bari, Piazza Giulio Cesare, 11-70124 Bari, Italy; marialisa.clodoveo@uniba.it (M.L.C.).

* Correspondence: mariamaddalena.cavalluzzi@uniba.it; Tel.: +039-080-5442736

Factor screening

In order to identify the factors that had the most significant effects on the extraction outcome as well as the values to be set in the subsequent optimization procedure (response surface methodology described in the manuscript), a 2-level full factorial design has been carried out using *Modde 6.0*. Three factors have been chosen: extraction temperature (58 °C and 68 °C), extraction time (5 and 10 min), and solid/liquid ratio (12.5 and 25 mg/mL, performing the extraction with 50 mg of bamboo shoots). The full factorial design included 8 experimental runs and 3 center point experiments (Table S1).

Table S1. Screening of parameters influencing microwave-assisted extraction of bamboo shoots: design matrix and observed responses.

Experiment No.	Extraction temperature (°C)	Extraction time (min)	Solid/liquid ratio (mg/mL)	TPC [a]
1	58	5	12.5	0.48
2	68	5	12.5	0.656
3	58	10	12.5	0.52
4	68	10	12.5	0.812
5	58	5	25	0.506
6	68	5	25	0.512
7	58	10	25	0.552
8	68	10	25	0.524
9	63	7.5	18.75	0.495
10	63	7.5	18.75	0.513
11	63	7.5	18.75	0.507

[a] expressed as mg GAE in the total extraction volume.

The model suggested that extraction time was a non-significant factor over the investigated ranges. Conversely, extraction temperature and solid/liquid ratio were determined to be significant factors.

As can be seen in Figure S1, the highest TPC content has been obtained at both higher temperature and solvent volume, thus indicating the direction for the best setting of these factors expected to yield even greater TPC values in the next optimization step.

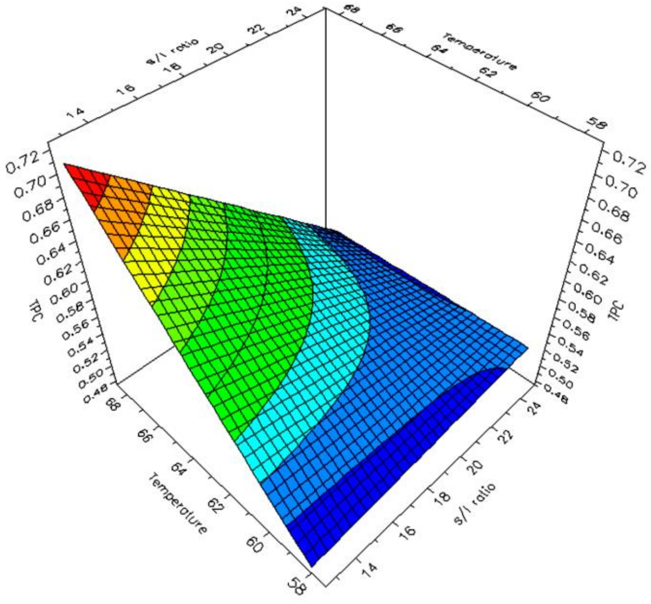

Figure S1. Response surface plot of the effect of extraction temperature and solid/liquid ratio on polyphenol yield (TPC) obtained through MAE.

Although the time variable was irrelevant in the initial screening phase and therefore we could have ignored it in the next phase of RSM, we tried to reduce it further and, therefore, we chose for the optimization step the following values:

- extraction temperature: 75 °C and 95 °C
- extraction time: 3 and 5 min
- solid/liquid ratio: 6.25 and 10 mg/mL.

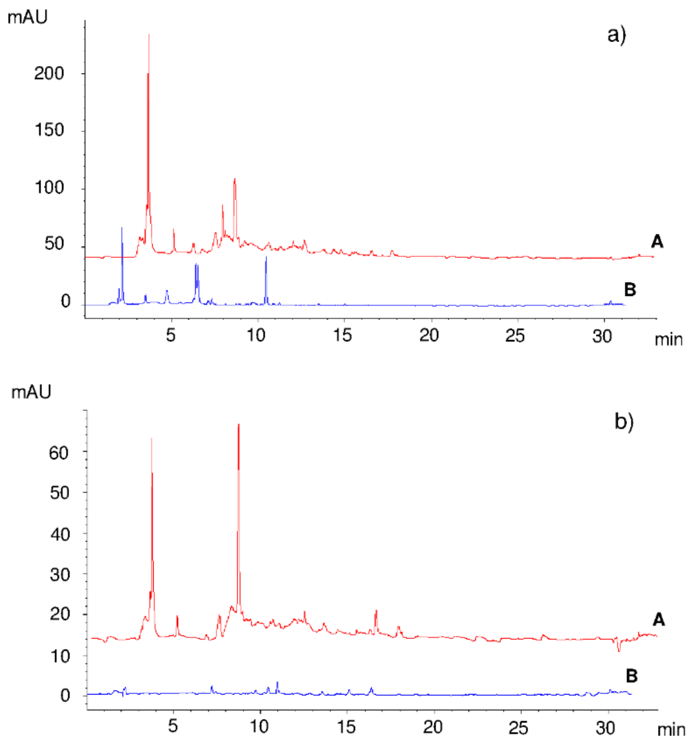

Figure S2. HPLC-DAD chromatograms recorded at a) 280 nm and b) 320 nm of the bamboo shoots MW extract obtained under optimal conditions (A) and conventional extract (B), respectively.
